# Educational Automatic Question Generation Improves Reading Comprehension in Non-native Speakers: A Learner-Centric Case Study

**DOI:** 10.3389/frai.2022.900304

**Published:** 2022-06-10

**Authors:** Tim Steuer, Anna Filighera, Thomas Tregel, André Miede

**Affiliations:** ^1^Multimedia Communications Lab (KOM), Technical University of Darmstadt, Darmstadt, Germany; ^2^Saarland University of Applied Sciences, Saarbrücken, Germany

**Keywords:** automatic question generation, self-assessment, natural language processing, reading comprehension, education

## Abstract

**Background:**

Asking learners manually authored questions about their readings improves their text comprehension. Yet, not all reading materials comprise sufficiently many questions and many informal reading materials do not contain any. Therefore, automatic question generation has great potential in education as it may alleviate the lack of questions. However, currently, there is insufficient evidence on whether or not those automatically generated questions are beneficial for learners' understanding in reading comprehension scenarios.

**Objectives:**

We investigate the positive and negative effects of automatically generated short-answer questions on learning outcomes in a reading comprehension scenario.

**Methods:**

A learner-centric, in between-groups, quasi-experimental reading comprehension case study with 48 college students is conducted. We test two hypotheses concerning positive and negative effects on learning outcomes during the text comprehension of science texts and descriptively explore how the generated questions influenced learners.

**Results:**

The results show a positive effect of the generated questions on the participants learning outcomes. However, we cannot entirely exclude question-induced adverse side effects on learning of non-questioned information. Interestingly, questions identified as computer-generated by learners nevertheless seemed to benefit their understanding.

**Take Away:**

Automatic question generation positively impacts reading comprehension in the given scenario. In the reported case study, even questions recognized as computer-generated supported reading comprehension.

## 1. Introduction

A crucial way of learning is by reading educational texts. They are omnipresent in our educational system, where students read textbooks, lecture notes, online resources, or worksheets. However, research suggests learning by passively reading texts is inefficient (Rouet and Vidal-Abarca, 2002). Students skim over facts, misunderstand ideas and comprehend only a fraction of the information provided. Considering how much time students spend reading, such inefficiencies caused by missing interaction may significantly impact their educational careers. Thus, actively engaging learners with their reading material is crucial to optimize their long-term learning trajectories. A well-established and intuitive way to foster learning is to pose questions about the reading material (Rickards, 1979; Hamaker, 1986; Rouet and Vidal-Abarca, 2002; Le et al., 2014). Such questions, explicitly inserted into the text to draw attention to the important textual material, are called adjunct questions (Dornisch, 2012).

**Figure 1:**
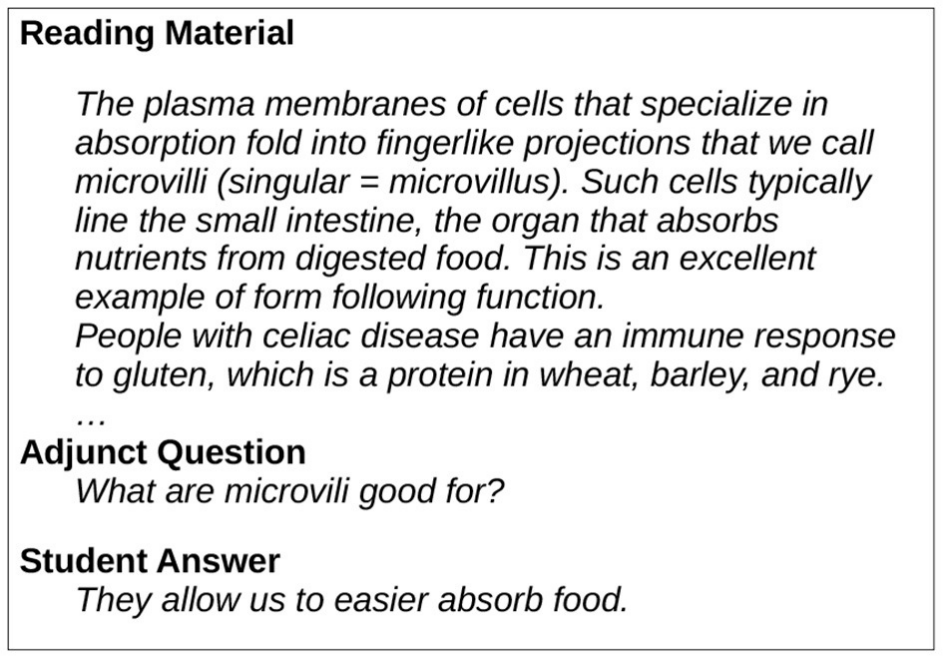
Adjunct literal questions for reading comprehension. Students answer questions after reading short text passages.

Although educational experts often consider deep comprehension questions more valuable (Hamaker, 1986; Rouet and Vidal-Abarca, 2002), manually authored adjunct literal questions are an established means to increase learning outcomes (see Figure 1) (Anderson and Biddle, 1975; Hamaker, 1986). Literal questions inquire about information directly presented in the text and involve only very limited inference or transfer processes. They cause various facilitative effects. They guide the learners' attention to essential or difficult ideas (Rickards, 1979)or trigger reflection of the consumed information, allowing learners to consolidate their comprehension (Rickards, 1979). Additionally, they may strengthen the mental memory trace of questioned information (Rouet and Vidal-Abarca, 2002) and not only foster learning of the facts in question but also closely related information (Hamaker, 1986). Finally, literal questions may be seen as a form of retrieval practice, inducing the testing effect (Roediger III and Karpicke, 2006), which improves long-term retention of the learnt information. This direct empirical evidence is complemented by reading comprehension theories stating that the readers' mental representations rely on conceptual networks (Kintsch, 1988; Rouet and Vidal-Abarca, 2002; Van Den Broek et al., 2002). In consequence, one could argue that literal questions support readers while structuring and navigating this mental representation by focussing their attention and orchestrating their concept retrieval (Rouet and Vidal-Abarca, 2002).

Educational AQG tries to alleviate the lack of manually authored questions in reading materials. And, while generating deep comprehension questions is still in its infancy, advances have recently been made in the generation of literal short-answer questions. Research has greatly improved literal question generators' fluency and naturalness on a wide variety of texts (Du et al., 2017; Wang et al., 2018). Given the ample empirical evidence and theoretical underpinning for the potential of literal questions, working AQG systems may provide significant benefits to learners and educators such as teachers.

On the educator side, operating AQG systems could save valuable time during the authoring process by providing a baseline of factual questions so that authors can focus on deep comprehension questions. For instance, higher education books are frequently written voluntarily or on a royalty basis by subject matter experts. Thus, authors will optimize their writing time and might neglect to author sufficiently many formative assessment questions for the text. In this example, AQG systems could support authors with a list of generated questions per book chapter. Authors only have to select a set of most relevant adjunct questions from the list, reducing their working time. In another example, teachers may employ reading materials for self-study before discussing the topic in class. They could generate literal questions *via* an AQG to ensure that learners recall the core concepts mentioned in the text, allowing them to focus their in-class discussion on deep comprehension questions.

On the learner side, AQGs may retrospectively improve informal or published texts by adding factual self-assessment questions even in scenarios where revising authors are absent, and automatic generation thus remains the only option. For instance, learners frequently rely on online resources such as Wikipedia. A student-facing AQG could support learners when working with such resources by recommending self-study questions for the articles' key concepts.

Yet, posing questions is an intuitive process. The exact procedure for asking valuable educational questions about texts is hard to formalize and involves more than generating fluent and natural texts. Consequently, automatically generating literal educational questions is still challenging. It usually encompasses the extraction of valuable educational facts (Content Selection) and their transformation into questions. In recent years, these challenges have been addressed by applying statistical and neural network-based algorithms to select important passages and concepts (Du and Cardie, 2017; Chen et al., 2019) and generate questions concerning their selection (Du et al., 2017; Dong et al., 2019).

Applying these approaches in educational contexts is promising, outperforming non-neural systems (Du et al., 2017; Wang et al., 2018). Moreover, systems trained on noneducational data generalize their language fluency to educational inputs (Wang et al., 2018; Steuer et al., 2020). However, initial expert-based studies show mixed results concerning the generated questions' educational value. Descriptive studies found questions with linguistic and pedagogical value (Steuer et al., 2020, 2021) . Experts agree on the linguistic quality characteristics. Yet, experts were in considerable disagreement about the questions' pedagogical value, measured by asking them whether or not they would use the question when teaching (Steuer et al., 2021). Horbach et al. (2020) have shown that experts rate the quality of generated literal questions as worse than the quality of manually authored questions. However, a question's educational value is hard to estimate, even for literal questions. Good educational questions are central to the text, cannot be misinterpreted and connect the learners' prior knowledge to their learning goals. Those quality characteristics depend on the learning arrangement, and estimating them is difficult even for experts (Amidei et al., 2018). For example, a simple literal question like “*Who was first president?”* could be beneficial for assessing learners' basic text understanding, even if it is ungrammatical. Depending on the text, it may be answerable or ambiguous and may achieve or miss the learning goals depending on the learners' goals, prior knowledge, and the text's content. Consequently, expert-based studies show that the annotators' opinions of what constitutes a valuable educational question often differ (Horbach et al., 2020; Steuer et al., 2021).

We are, therefore, interested in the learner-centric view and the potential positive and negative effects of educational AQG during reading comprehension. We are curious if the literal generated questions increase or decrease learning outcomes. Until now, this association has mostly been estimated indirectly *via* expert studies with no conclusive result. Additionally, we are interested in the relation between the learners' perception of the questions' author (human/computer) and the associated learning effects. So far, expert-based evaluations often assume the more human-written a question sounds, the better the question, and we wonder if this assumption holds in an actual learning scenario. We derive the research question:

RQ:To what extent do adjunct questions influence learners' text comprehension?

We seek to answer the question by conducting a learner-centric, in between-groups, quasi-experimental reading comprehension case study with *N* = 48 participants. The study was conducted with English materials due to the high availability of NLP techniques for the English language and recruited mainly subjects with advanced English proficiency. For the remainder of the article, we will use the phrase *adjunct question* to refer to literal short-answer questions generated by an educational AQG used for self-assessment purposes after reading text passages.

This report's main contributions encompass:

- Results from hypotheses tests providing evidence that automatically-generated questions positively affect the learning of information related to the question.
- A descriptive analysis suggesting that automatically-generated questions frequently fool learners into judging them as manually authored.
- A descriptive analysis showing that automatically-generated questions supported learning even when users identified them as machine-authored.

## 2. Related Work

Our discussion of the related work starts by establishing that manually authored questions influence and foster learning. Next, an overview of AQG methods is given. Different AQG and content selection approaches and their limits are discussed. Last, we discuss the evaluation of AQG systems and what is currently known about their question quality.

### 2.1. Effects of Manual Questions on Learning

There is a large body of evidence establishing questioning as an effective way to foster learning. One of the first large-scale reviews on the effects of adjunct questions for text comprehension by Anderson and Biddle (1975) examined the impact of questioning on reading comprehension. The authors surveyed 79 studies, with 63 studies finding positive effects of questions posed after a small paragraph. Furthermore, the authors distinguished between experiments repeating the intervention questions verbatim in the posttest and experiments that asked novel questions. Scores on the repeated posttest questions were significantly improved in the reviewed studies. While most surveyed studies also found an effect for the novel posttest questions, the authors could not reproduce these findings. In their experiments, paraphrase questions did not result in better scores than verbatim questions. However, participants of the paraphrase condition remembered more in a 1-week delayed posttest. Moreover, different questioning formats were reviewed. Short-answer questions exceeded multiple-choice questions in terms of improvement over the control group.

Another review was conducted by Hamaker (1986). The study included 61 adjunct question experiments, some overlapping with Anderson and Biddle (1975). Hamaker (1986) estimated the included studies' mean effect to approximately one sigma on the effect size scale by Bloom (1984). The average increase in performance was 37.5% relative to the control conditions. The survey distinguished between experiments with repeated and novel posttest questions and introduced a third category: related posttest questions. Those questions are not directly repeated, but answering them requires similar learning activity as answering an adjunct question. The authors found that repeated questions had the most substantial mean effect on posttest performance. Related posttest questions were also effective, and unrelated questions had no effects. The findings have been validated and discussed from various angles in the following years (Roediger III and Karpicke, 2006; Callender and McDaniel, 2007; VanLehn et al., 2007). Today, we have ample evidence for questioning fostering understanding in many circumstances (Rouet and Vidal-Abarca, 2002).

Besides direct empirical evidence, theories explaining why questioning is a helpful learning activity developed. Theories like the construction-integration model (Kintsch, 1988), the QUEST model of question answering (Rouet and Vidal-Abarca, 2002) or the Landscape model (Van Den Broek et al., 2002) emerged. They model reading comprehension as activation processes of different concepts and their relations in the reader's mind. Answering a question causes search processes for the conceptual network needed to answer the question and its corresponding activation. Put differently, questioning guides the attention and scaffolds retrieval practices of the information in question. Consequently, it becomes more likely that the learner successfully remembers the information.

In summary, the related work provides solid evidence for the positive effects of manually authored short-answer questions. They foster learning and retention. It is thereby vital that the question is related to the information that should be learned. Additionally, questions have attention guiding effects that may influence what will be learned and forgotten.

### 2.2. Automatic Question Generation in Education

In recent years, natural language generation mainly advanced through statistical learning (Radford et al., 2019). The models mimic the regularities of language to produce human-like texts. Consequently, similar models have been explored in AQG. Sequence-to-sequence models emerged, transforming declarative sentences into questions. They were more effective than previous methods (Du et al., 2017). Further improvements were achieved by applying pre-trained transformers (Dong et al., 2019) or different optimization goals (Qi et al., 2020). These neural network-based models have been transferred to educational AQG. Initial research suggests that the linguistic strengths of these models carry over to educational datasets (Wang et al., 2018; Steuer et al., 2020).

However, the educational setting is more challenging because asking about random facts is inexpedient. Instead, educational AQG requires learning-relevant questions. Thus, educational neural question generators must be combined with a content selection technique, extracting learning-relevant information. Initial content selection approaches assumed that a generic summary also includes many learning relevant facts (Chen et al., 2019). Hence, they apply text summarization algorithms as content selectors. Yet, no algorithm performs well on all educational datasets (Chen et al., 2019). Data-centric, specialized approaches try to learn what constitutes learning-relevant information from given educational data, and they outperform non-specialized content selection methods (Subramanian et al., 2018; Willis et al., 2019). However, in such systems, the data implicitly defines what constitutes relevant content for posing a question. The machine learning models then learn the implicit definition of learning-relevant content from the data. Thus, indicating what the trained system will regard as question-worthy given a specific sentence is almost impossible a priori because the learnt selection criteria from the data are never explicated. This limits the model application because the transferability of the systems to unseen domains is unknown. Moreover, even if the approaches function well, their underlying learned pedagogical considerations cannot be explained. After all, we do not know which implicit criteria the system has learned to consider as an indicator for question-worthy information. Finally, some approaches select the content based on educational priors (Pavlik et al., 2020; Stasaski et al., 2021; Steuer et al., 2021). In other words, they work with an underlying educational theory or assumption explaining why specific textual passages should be considered question-worthy. They then built their content selection approach on this theory or assumption. For example, (Stasaski et al., 2021) explicitly built on the educational prior that causal sentences are learning-relevant and extract them *via* semantic role labeling. Such approaches are advantageous because their underlying pedagogical considerations are well defined, can easily be explained and are likely to transfer to novel data.

### 2.3. Evaluation of Automatic Question Generation

The evaluation of AQG systems is challenging. Linguistic quality is frequently estimated by automatic measures such as BLEU (Du et al., 2017; Qi et al., 2020) or by annotation studies (Du et al., 2017; Horbach et al., 2020; Steuer et al., 2020). Yet, automatic scores often only correlate weakly with a human judgement of linguistic question quality (Liu et al., 2016; Steuer et al., 2020). Furthermore, annotation studies are complex to conduct, as it is difficult for raters to agree on what constitutes a good question in various dimensions (Amidei et al., 2018; Horbach et al., 2020). Besides evaluating linguistic quality, educational AQGs must also be evaluated in their application context. Currently, the evidence of whether or not educational AQG could be helpful for learning is inconclusive. Results, for example, suggest that educational AQGs are favorable to noneducational AQGs (Wang et al., 2018). Other studies find that the general educational value of the questions is still limited (Horbach et al., 2020) and that the questions are mainly focused on non-central facts (Steuer et al., 2020). Moreover, many studies report promising results based on expert ratings but do not investigate their generated questions in a learner-centric study (Wang et al., 2018; Stasaski et al., 2021; Steuer et al., 2021). However, these expert evaluations have limitations. Many aspects that constitute educational values are hard to agree on by experts (Amidei et al., 2018; Horbach et al., 2020), and some learning-relevant dimensions can only be measured in an explicit educational setting (Stasaski et al., 2021; Steuer et al., 2021).

Some studies applied learner-centric evaluation. For instance, Lu et al. (2021) found that non-neural educational AQG improves the learning of programming concepts. Furthermore, Van Campenhout et al. (2021) investigated AQG for fill-in-the-blank questions generated on textbooks in a massive open online course scenario. Generated items had similar characteristics to manually authored questions regarding difficulty, engagement and students' answer persistence. Additionally, learners did not prefer manually authored questions over machine-generated questions. Finally, (Syed et al., 2020) compared the effect on learning outcomes of an eye-tracking-based short answer AQG, a no questions condition and having manually authored questions. In their study, the generated questions had positive effects and outperformed the manually authored questions in posttest scores.

In summary, neural network-based AQG has advanced the state-of-the-art in terms of linguistic quality. They have to be combined with educationally valuable content selection algorithms. There are many content selection methods currently explored in educational AQG, and we believe that approaches based on educational priors are the most promising. The expert-study evaluations of the systems are inconclusive and have limits. Yet, the few studies investigating educational AQG in a learner-centric setting are promising.

## 3. Technical Approach

To investigate the given research question, a AQG system has to be chosen. In this work, we rely on the system by Steuer et al. (2021). The system generates literal questions about the definitory content of a single chapter or whole textbook. Definitory contents are, for instance, the sentences:“*Intermediates of dsRNA, called replicative intermediates are made in the process of copying the genomic RNA.”* or “*A variable is any part of the experiment that can vary or change during the experiment.”* We select it for the following reasons.

First, it applies content selection using sound educational priors, relying on the assumption that definitory content is usually learning-relevant (Graesser et al., 2010). This assumption leads to a precision-oriented content selection approach. It is geared toward question quality instead of question quantity by focusing on automatically detecting definitions ignoring all other text content. Consequently, it is less likely to generate questions about learning-irrelevant text content, but it also misses relevant content. We believe that this precision-oriented approach is vital for AQG systems. In Human-Computer Interaction research, it has been shown that failures causing the most harm to the user should be minimized (Kocielnik et al., 2019), and we assume that irrelevant questions are significantly more harmful to the user than possibly missing questions. Hence, a focus on high precision generation is critical.

Second, definitions are present in various text forms and follow general language patterns relatively independent of the text's domain. Accordingly, machine learning algorithms can classify sentences as definitions stemming from numerous domains (Spala et al., 2020). Therefore, when compared to systems directly learning the distinction between relevant and irrelevant information on a training corpus, definition classification has advantages for AQG content selection. On the one hand, the question relevant information is defined a priori because only definition phrases are relevant. If, on the other hand, question relevance is learned from the data, it becomes intricate to know for which criteria the algorithm selects relevant information and whether the learned criterion is didactically meaningful. Moreover, it is easier to estimate if the content selection by definitions will function for a given text because humans can apply a similar selection criterion as the machine learning algorithm was trained for.

**Figure 2:**
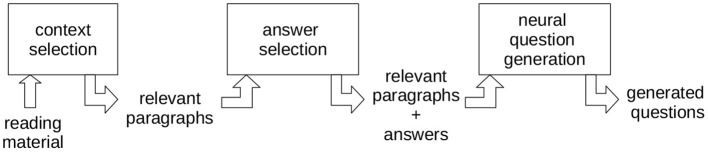
An overview of the involved steps in the employed AQG approach.

Third, the system has shown interesting performance characteristics in an expert study examining the generated questions' linguistic and pedagogical quality. Most questions were rated favorably regarding linguistic quality, whereas the pedagogical rating was diverse, comprising considerable disagreement. We thus believe that a learner-based evaluation may shed more light on the educational capabilities of the adjunct questions. Technically, it comprises three subphases: context selection, answer selection and neural question generation (see Figure 2). We will give a brief overview of its core mechanisms and refer to Steuer et al. (2021) for an in-depth technical description and expert annotation study.

During context selection, the system determines a subset of the given text as the question generator's context window. For instance, let's assume a textbook chapter with thirty pages. Then, the subset selected will only consist of a couple of essential paragraphs. Essential paragraphs contain definitory content and also mention concepts from the back-of-the-book index. This ensures that the selected paragraphs fit into the question generator's input boundaries and that the inputs for every question are monothematic. The text is filtered for indexed concepts, only retaining candidate sentences where the concepts are mentioned. The candidate sentences are classified using a binary classifier. Either a candidate sentence is a definition or not. The system relies on a DistilBERT (Sanh et al., 2019) classifier trained on the large-scale DEFT corpus (Spala et al., 2019).

Additionally, a task-specific adaptation of the classifier is applied, setting the decision threshold to 0.70. The idea is to favor classification errors that affect the users the least (Kocielnik et al., 2019). Hence, it will generate fewer questions, but the questions will more likely originate from an actual definition. Classified definitions will be used to contextualize the question generator.

Second, given the context, the approach selects an educationally valuable answer inside the context (Answer Selection). The answer is necessary to limit the generator's degrees of freedom as grammatically, many questions about a context are possible, yet most are irrelevant for learning. The context paragraphs contain definitions, and thus promising answers consist of the concept that will be defined or the specific characteristics stated to explain the concept. The approach uses pattern matching over the dependency graph of the sentences to extract sentence subparts describing essential characteristics of the concept. The patterns are, for instance, able to extract relative clauses which characterize specific concepts in a sentence. Depending on the complexity of the input sentence, multiple patterns match. In these cases, the longest matching phrase is selected, assuming the longer a phrase, the more meaning it conveys.

The selected answer and the context become the input of the neural AQG. The neural question generator produces questions conditioned on the answer and context provided using the answer-aware approach by Dong et al. (2019). It applies greedy text decoding. The final output of the system consists of questions about the definitions in a given text together with their potential answers.

## 4. Methods

### 4.1. Study Design

We conducted a learner-centric, pre-/posttest reading comprehension quasi-experiment in a between-groups design investigating the research question of the introduction:

RQ:To what extent do adjunct questions influence learners' text comprehension?

We focus mainly on the influence of the adjunct questions on the learning outcome in contrast to a no-question control group. The learning outcome is central to estimate the success of an educational technology. However, the related work usually does not measure it directly but relies on expert annotation studies. The studies measuring it, did apply either non-neural AQG approaches (Lu et al., 2021) or incorporated other factors apart from textual ones (Syed et al., 2020).

We distinguish between learning outcomes on topics directly related to the previously read adjunct questions (related learning outcome) and unrelated to the adjunct questions (unrelated learning outcome). The related work shows that manually authored adjunct questions only foster related learning outcome but not unrelated learning outcome (Hamaker, 1986). Moreover, adjunct questions presented before reading materials may have an attention priming effect that possibly affects the learner negatively. The review by Hamaker (1986) reported such negative effects for pre-reading material adjunct questions. Yet, no negative effects could be found for adjunct questions presented after the reading material (Hamaker, 1986). We would therefore expect an increase in related outcome and no decrease in unrelated learning outcome. However, we do not want to rule out a decrease in unrelated learning outcome a priori because the adjunct questions are not manually authored. Due to their varying quality, their negative effect on learners may be more pronounced. Consequently, we will test the following hypotheses:

H1 The treatment group will have a higher related learning outcome compared to the control group
H2 There is no difference between the treatment and control group in the unrelated learning outcome

Accompanying the hypothesis testing, we aim to understand if and why the adjunct questions impacted the learning outcome. Thus, we capture additional variables (see Table 1). We measure the perceived author of the adjunct questions through self-report (computer/human). Many expert-based studies assume that the more a question sounds human-written, the better it supports learning. Hence, it should be a good summary measure, correlating with various quality characteristics considered necessary by expert studies. It captures elements of linguistic quality such as the grammaticality, naturalness, and fluency of the adjunct questions. It also captures pedagogical characteristics such as the questions' connectedness to the text and learning relevant concepts. Furthermore, if the assumption of the expert studies is valid, adjunct questions perceived as human-written should correlate with an increase in learning outcomes. We additionally record the answers to every adjunct question and measure the confounding variables of prior “knowledge, English language skills, demographic factors and time-on-task.

**Table 1:** Variables elicited in the experiment.

**Type**	**Variable**
Independent	Adjunct question condition (yes/no)
Dependent	Related learning outcome
	Unrelated learning outcome
Other	Time-on-task
	Language skill
	Prior knowledge
	Student answers to the adjunct questions
	Adjunct questions' perceived author (computer/human)

Our explorative analysis is guided by the question:

E1 How did the perceived author (human/computer) and answerability of the adjunct questions influenc e learning outcomes?

We operationalize answerability as whether or not participants perceived the question as answerable. Thus, a question is unanswerable if participants indicate that they cannot provide an answer and is considered answerable if they provide an answer. Note that we do not check the answer quality (e.g., correctness).

### 4.2. Participants

We sampled participants with three different procedures. First, roughly a third of the participants were sampled through a single university course. They were contacted during an online lecture and could participate voluntarily in the study. We incentivized their participation with a lottery of Amazon gift cards (5 x 20€) and exercise bonus points to increase the exam grade. We also offered an alternative exercise to collect the bonus for students who did not want to participate. Second, another third of participants were recruited *via* email mailing lists and advertising in various online courses of a different university. We used the lottery as an incentive for those people. Third, some participants were sampled over Facebook survey circles. People cooperating in a survey circle will participate in an experiment if someone takes part in their experiment in return. They were thus incentivized through additional participation in their experiment and were also allowed to participate in the lottery.

We are aware that the sampling procedures applied relied on recruiting participants through external rewards. Thus, it is not unlikely that some of the participants had no honest intention to participate. Instead, they may try to game the system to get their reward. Thus, we showed three control questions during the experiment to detect cheating attempts. Incorrectly answering one of these questions led to the exclusion from the experiment. A total of 57 participants finished the experiment. After filtering for cheating attempts through the control items, *N* = 48 participants remained, randomly assigned into equally sized control and treatment groups of 24 participants. The sample comprised 17 females, 30 males and 1 non-binary with an average age of *M* = 24 *years, SD* = 3.60 *years*. The participants reported their language levels according to the Common European Framework of Reference for Languages (CEFR) levels. It ranged from A2 (elementary) to C2 (mastery). However, 83% of all participants spoke English on a level of B2 (upper-intermediate) or better. Most participants studied at a university between 1 and 15 semesters, with most people in their second semester (*N* = 17). Only four participants have already completed their studies. The participants studied computer science or related (*N* = 34), electrical engineering or related (*N* = 12) or psychology or related (*N* = 2).

### 4.3. Material

The used material for the experiment encompassed two reading comprehension texts and posttests. Prior knowledge of the texts' content was measured *via* self-report. We rely on self-reporting measures instead of a pre-/posttest design because pre-test questions likely prime the participants' reading attention toward specific information in the text. Consequently, participants would pay less attention to information not covered by the pre-test (Hamaker, 1986), which would result in a severe threat to the validity of the reading comprehension study. Moreover, the material was deliberately chosen so that the sampling procedure will likely yield participants with very little prior knowledge. Hence, although we know that self-reporting has limitations and is not the most accurate measure of prior knowledge, we assume it is justified to rely on it for this study because it alleviates priming effects and still provides a proxy measure for potential prior knowledge group differences.

The reading comprehension texts concerned Eukaryotic Cells (Biology) and the Layers of the Skin (Anatomy). Both texts were initially taken from OpenStax books^1^ and were revised to be self-contained. We have chosen the topics as they represent contents a typical undergraduate course covers and contain sufficiently many novel concepts that must be defined. The material included the text and all figures used in the chapter. The biology text had a length of about 1,400 words, and the anatomy text had about 1700 words. Both texts were split into three logical passages. We generated two adjunct questions for every passage resulting in six questions per text. The adjunct questions were generated offline before the experiment. All words in bold-face served as index concepts of the two texts. Before using the questions in the experiment, we applied a quick readability check but did not control their text relatedness, answerability, or other factors. For a single passage, the two initially generated adjunct questions were clearly incomprehensible. These questions were regenerated once again with a different random seed.

We had two separate posttests for biology and anatomy. Each posttest consisted of 12 manually authored single-choice items with four distractors each. Six postest items measured the participants' related learning outcome. The other six posttest items measured the participants' unrelated learning outcome. The test furthermore comprised the three control items to detect cheating attempts. The related learning outcome posttest items were generated after the adjunct questions to ensure their relatedness. They were no verbatim copies of the adjunct question. Instead, they paraphrased an adjunct question or covered closely related information. Three revisions have been applied to the material before it was used in the study. These revisions did not affect the generated questions, as they were generated initially and did not receive further manual revision. During every revision, between three and ten experts worked with the material and gave feedback. As a result of these revision cycles, we rephrased ambiguous instructions, exchanged unfitting posttest items and distractors, and enhanced text readability.

### 4.4. Procedure

**Figure 3:**
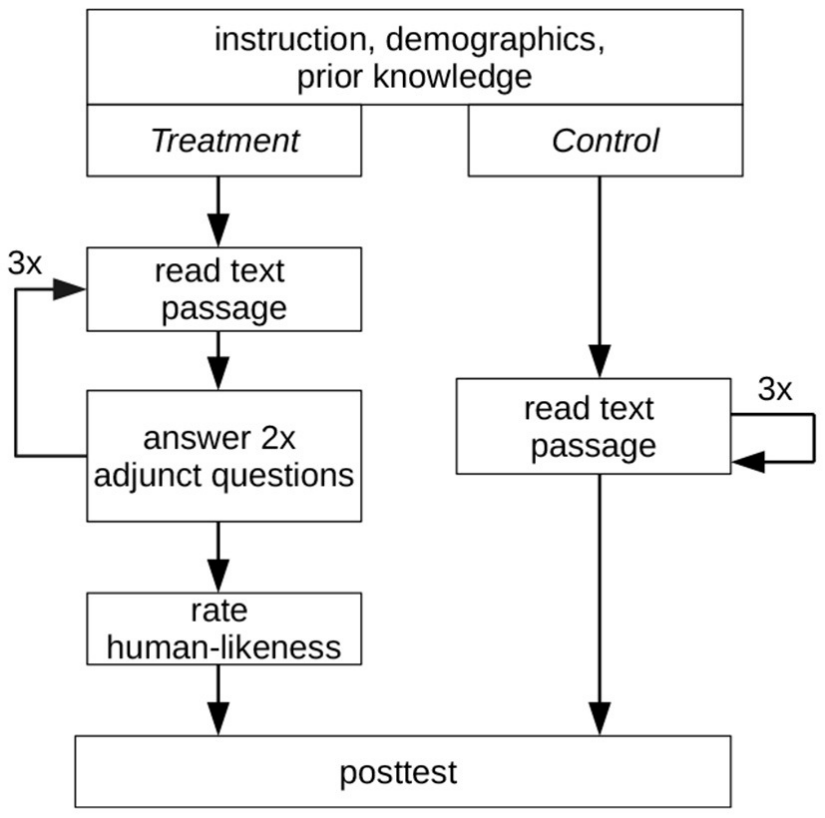
An overview of the experimental procedure followed. Each group read three text passages. Additionally, the treatment group answered two adjunct questions after every text passage.

The experiment ran over 6 weeks entirely online (see Figure 3). First, a link to the experiment was distributed *via* the learning management system, email or Zoom videoconferencing. Besides the link to the experiment, the message contained a soft deadline of 2 weeks to nudge the participants into timely participation. Furthermore, the message detailed the incentives and that participants need roughly 45 min of quiet working conditions to participate successfully. Second, after visiting the link, the system automatically assigned every participant randomly to one of four conditions based on the treatment or control group and the reading material used (Biology vs. Anatomy). After opening the survey link, the task was summarized on a web page, and the participation requirements were given. Thus far, participants could still postpone participation. At the start of the experiment, participants were requested to give their demographical information. They were reminded to be diligent and to not cheat. It followed a self-report of their prior knowledge to alleviate priming effects (see Section 4.3). We used a five-point Likert scale with five items, where participants rated their knowledge from 0 (*know nothing*) to 4 (*expert*). Next, detailed instructions on how to approach the reading comprehension were given. In general, they were informed that the readings are complex and likely unfamiliar, but that this is on purpose. We allowed the use of two online translation services to translate unknown words. Furthermore, they were reminded that a posttest follows the reading comprehension, that cheating is not allowed and that they cannot return once they have started the reading comprehension. Treatment group participants were informed to answer all adjunct questions with two or three sentences in their own words. Furthermore, we instructed the treatment group on how to indicate incomprehensible adjunct questions. This was followed by the reading comprehension part. The respective reading material (biology or anatomy) was divided into three text passages of similar length, shown consecutively. The control group read one passage after another. Participants could determine their pacing on their own but were instructed only to proceed when they learnt the information on the page. The exact text passages were also used for the treatment group, but two adjunct questions were shown after each passage. Each adjunct question required a short text answer from the participants. Participants could not skip back and forth between the text passages. After the reading comprehension, the treatment group was asked to rate the adjunct questions as either manually authored or computer-generated (binary scale). Finally, the posttest and a final information page concluded the experiment for both groups. The information page allowed participants to provide their email addresses for the lottery incentive and to remark if they were interrupted during the experiment. A debriefing describing the experiment and its measures was done for participants sampled in the lecture. For the other participants, we offered the option to contact us for a debriefing session *via* email, an opportunity used by one participant.

## 5. Results

We will give an in-depth analysis of the dataset's characteristics. Additionally, we provide the results of the hypothesis tests concerning the learning outcomes.

### 5.1. Explorative Analysis

The 48 participants are evenly split among control and treatment conditions. Inside each condition, participants saw one of two reading materials (biology or anatomy). The biology text has been read by 10 participants in the treatment group and 11 participants in the control group. The anatomy text has been read by 14 participants in the treatment group and 13 participants in the control group. Thus, the anatomy text has been read more often than the biology text. No ceiling or floor effects occurred in the different texts' posttest items. The mean item scores range from *Min* = 0.16 to *Max* = 0.80 in anatomy and *Min* = 0.44 to *Max* = 0.83 in biology. We will collapse the reading material factor by combining the data of both reading materials for the rest of the analysis. We do this as our sampling did not yield enough data points for an in-depth analysis of its influence. As a result, we have 24 participants in the control group (*M* = 24.38 *years SD* = 3.15 *years*) and 24 participants in the treatment group (*M* = 23.67 *years SD* = 4.08 *years*).

We normalize all reported testscores to be between zero and one using the following formula:


(1) $$\begin{array}{r} score_{test}=\frac{\sum score_{q}}{score_{max}} \end{array}$$

In the posttest every right answer awards one point. The corresponding *score*_*max*_ are 12 points for the posttest. Thus, if a participant scores 3 out of 12 correct, we achieve a *score*_*test*_ = 0.25. The same normalization procedure is used for participants' self-reported prior knowledge with *score*_*max*_ = 20. Moreover, the scheme is also used to score participants' language skills, with skill A1 corresponding to zero points and C2 corresponding to five points.

We begin by reporting the distribution of potential confounding factors. The participants' language skills are almost evenly distributed with a mean score of *M* = 0.71 *SD* = 0.20 in the treatment group and *M* = 0.68 *SD* = 0.19 in the control group. The participants' prior knowledge also varies little across groups with a mean score of *M* = 0.30 *SD* = 0.22 in the treatment group and *M* = 0.28 *SD* = 0.21 in the control group. The time-on-task for the treatment group *M* = 34 *min SD* = 13 *min* is higher than for the control group *M* = 29 *min SD* = 14 *min*.

**Figure 4:**
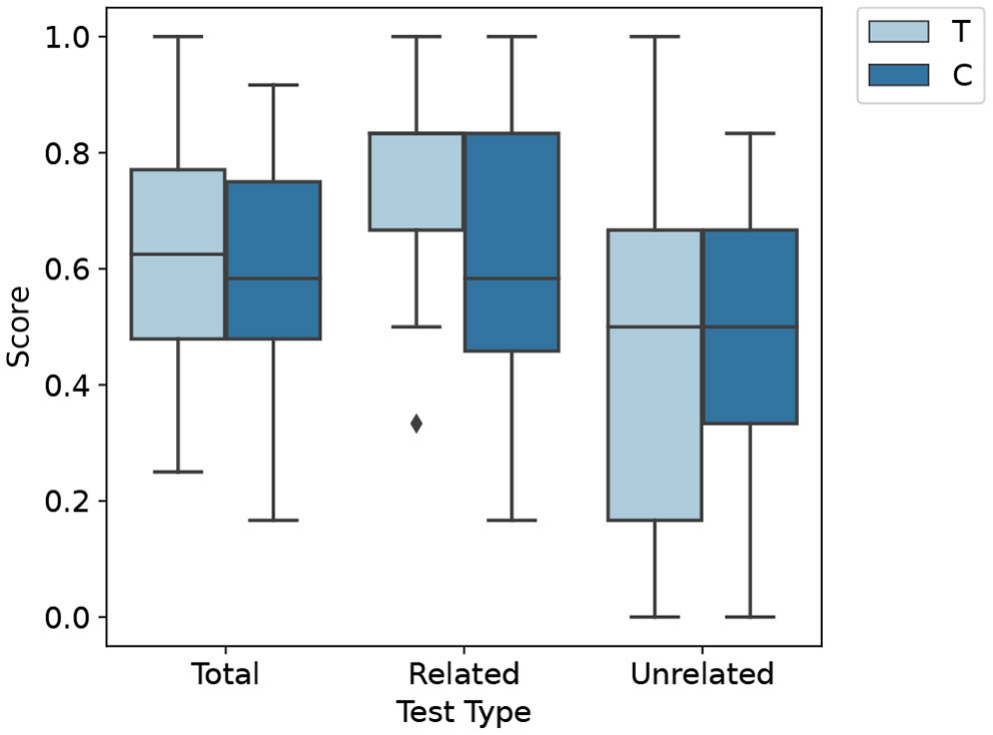
Boxplot of the different test scores of the treatment (T) and control (C) groups. Whiskers indicate 1.5 interquartile range and the bar inside the box indactes the median. The median of the *Related* group is at the top of the box.

**Figure 5:**
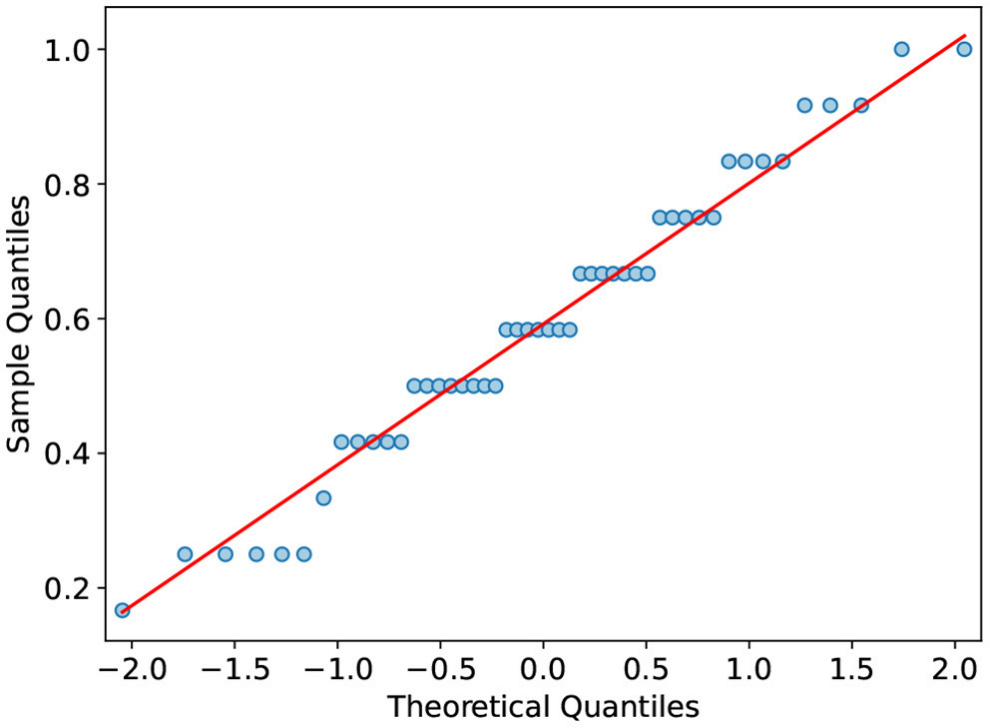
Normal quantile-quantile plot of the total posttest scores. The red line visualizes a normal distribution for comparision. The x-axis shows the z-scores of the normal distribution. The y-axis shows the total posttest score.

Next, the distribution of the posttest scores is summarized. The boxplot visualizes the distribution of the various test scores (Figure 4). All scores overlap substantially. The related posttest score differs the most between groups. Additionally, it has the narrowest distribution in the treatment group. On average, the control group has a lower total score in the posttest with *M* = 0.56 *SD* = 0.21 compared to *M* = 0.62 *SD* = 0.22 of the treatment group. The difference in the total posttest score is mainly due to the control group performing worse in terms of related learning outcome (*M* = 0.62 *SD* = 0.25), whereas the treatment group performs better (*M* = 0.76 *SD* = 0.19). There is only a slight difference in terms of unrelated learning outcome (*M*_*C*_ = 0.51 *SD*_*C*_ = 0.25 *M*_*T*_ = 0.49 *SD*_*T*_ = 0.30). The total posttest scores' Quantile-Quantile plot (Figure 5) shows that the scores are not strictly normally distributed. It furthermore comprises line artifacts due to few distinct scores achievable.

Finally, we report the results on the perceived author judgement and on the meaningful answering of the adjunct questions. Both were only gathered in the treatment group. Participants perceived the adjunct questions overall as answerable. Only in 6 of 144 interactions, a question was rated incomprehensible. Five of the six interactions involved the same question (see Table 2 - question 3). The answers given by the participants were usually short paraphrases of the text content. The responses varied in their wording and content, although some questions provoked similar answers by inquiring a single concept. All responses were honest answer attempts. In addition, the participants estimated, on average, half of all generated questions as manually authored *M* = 2.95 *SD* = 1.12, *Min* = 1, *Max* = 6. No question was identified as computer-generated by all participants. The distribution of the different perceived author ratings can be seen in Table 2. The question rated the most as human-written was only rated once as computer-generated, while the least received nine votes. The incomprehensible question discussed previously was rated eight times as computer-generated and six times as manually authored.

**Table 2:** The 12 adjunct questions used in the experiment and their mean perceived author rating.

**No**	**Question**	**Perceived as human-written**
1	The skin and its accessory structures make up what system?	0.43
2	What are cells in all of the layers except the stratum basale called?	0.57
3	What happens to the growth of fingerprints in a growing fetus?	0.43
4	How are keratinocytes formed?	0.71
5	What does the hypodermis serve?	0.36
6	What cells produce melanin?	0.57
7	What does the plasma membrane control?	0.30
8	What is the structure of the cytoplasm?	0.90
9	Where is the nucleoplasm located?	0.80
10	What does Chromatin describe?	0.20
11	What do scientists often call mitochondria?	0.20
12	What are mitochondria?	0.40

*A high rating indicates that the question is perceived more often as human-written*.

**Figure 6:**
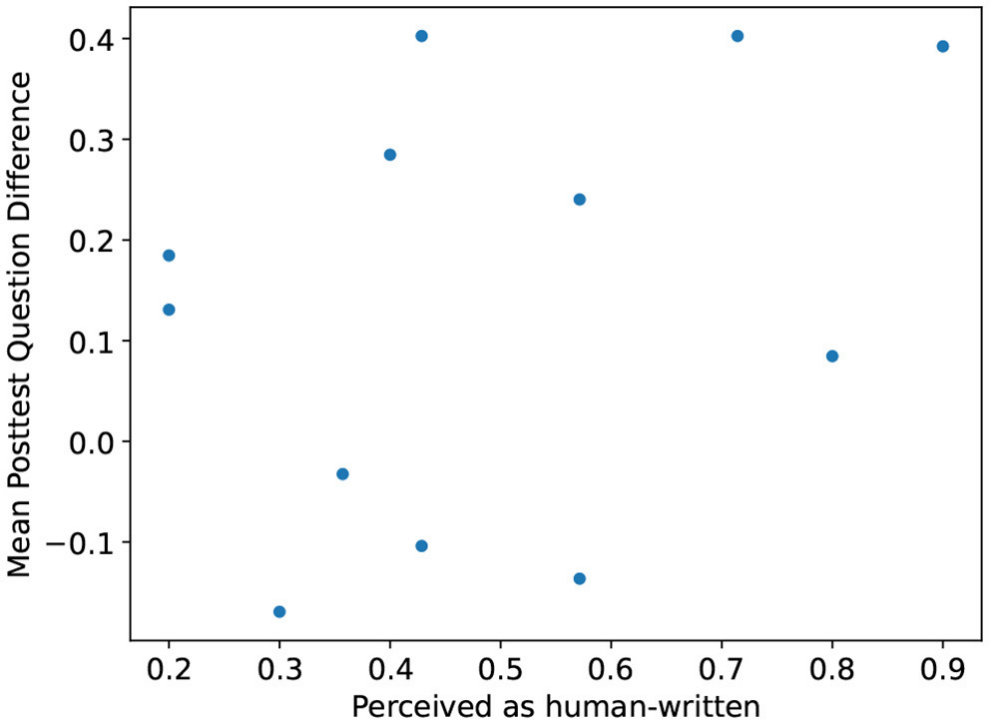
Scatterplot of the related learning outcome and the mean questions' perceived author. The y-axis indicates the mean difference in the correct answer ratio for the control and treatment groups of every posttest item. The x-axis shows their mean perceived author rating. The higher the rating the more participants rated the question to be human-written.

Although confounded by other factors, we hypothesized there might be a connection between the perceived author rating and the performance of participants on the corresponding related posttest item. In other words, we assume, that the more a question is perceived as human-written, the more likely it increases the participants' performance. Hence, we computed the mean difference between the control and treatment groups' correct answer ratios for related posttest items in biology and anatomy. An increase in mean difference points toward helpful questions. Next, we scatter plotted the mean difference, and the corresponding mean perceived author ratio (see Figure 6). The plot comprises twelve data points, one for every related posttest item. In the plot, positive y-values indicate an increase in the item's posttest score of the treatment group over the control group and vice versa. The x-values indicate how frequently a question was rated to be produced by a human. We suspected that the more often a question is considered human-written, the better understandable, the higher its mean posttest difference. Interestingly, there was no clear correlation detectable between the variables.

### 5.2. Evaluation of the Hypotheses

Considering the first hypothesis, we compare the learning outcome of the six related posttest items in the control and treatment groups. Our null hypothesis assumes no related learning outcome difference between groups, with a significance level of 5%. We use the Mann-Whitney-U test to test for statistically significant differences in the groups because the data is not normally distributed. The Mann-Whitney-U test indicated that the related posttest score was significantly higher for the treatment group (*Mdn* = 0.83) than for the control group (*Mdn* = 0.58, *U* = 194.5 *p* = 0.049). The effect size is Cohens *d* = 0.63 indicating a medium effect (Sawilowsky, 2009). On the effect size scale of Hamaker (1986), it corresponds to an effect size of *ES* = 0.14.

The second hypothesis concerns the unrelated learning outcome in both groups. Our null hypothesis is that there are no significant differences between groups. We apply the Mann-Whitney-U test adapted for equivalence testing as described in Wellek (2010). Conceptually, the test is the nonparametric pendant to the two-one-sided *t*-test (TOST) procedure, testing whether the two means differ by no more than ϵ to either side. We use liberal tolerances of ϵ = 0.20 on both sides as recommended by (Wellek, 2010). The test does not provide significant results at the α = 0.05 level between the control group (*Mdn* = 0.50) and the treatment group (*Mdn* = 0.50) with *W*+ = 0.45, σ = 0.08, *crit* = 0.08.

Given the result, we are interested in whether the control group performs better than the treatment group. We apply a directional Mann-Whitney-U test to the unrelated posttest scores at α = 0.05 level. The null hypothesis is that the control group scores higher than the treatment group. The test is also non-significant (*U* = 0.26 *p* = 0.40). Hence, neither equivalency nor control group superiority is statistically significant on the chosen significance level. Finally, although our results are not strictly t-distributed, we compute the 95% confidence interval of the means to contextualize our findings. The interval ranges from [0.40, 0.61] in the control group and from [0.36, 0.61] for the treatment group.

## 6. Discussion

We will begin our discussion with the hypotheses tests. Hereafter, we connect exploratory data analysis and hypothesis-testing results. We conclude the discussion by deducing possible implications for educational AQG from our findings. Furthermore, the section will address some limitations that might affect the validity of our conclusions and closes with an outlook on future work.

### 6.1. On the Positive Effect of the Adjunct Questions

The hypothesis (H1) postulates a positive effect of the adjunct questions on the related learning outcome. It is supported by the data with a medium effect size. Furthermore, the confounding factors prior knowledge and language skills in the different experimental groups are similarly distributed. The factor time-on-task is higher for the treatment group. Unsurprisingly, as it by definition mediates the adjunct questions' effect. Learners engage in more depth with the text to answer the adjunct questions increasing time-on-task. Nevertheless, this prolonged participation time may also affect learners negatively. It may have caused a fatigue effect, where subjects become tired of participating in the experiment, diminishing their performance. Assessing the presence of the fatigue effect is non-trivial and out of the scope of this case study. However, it is an interesting opportunity for future work as this may even increase the effect of the adjunct questions.

Based on the evidence, we accept hypothesis H1. In other words, the treatment group outperformed the control group in terms of related learning outcome. The finding is consistent with the effect of manually authored questions and shows that AQG systems can be an effective means to support learners. In terms of the magnitude of effect, Hamaker (1986) reported a mean effect size of 0.16 measured by exactly repeated posttest items and a mean effect size of 0.07 for studies involving related posttest items. In comparison, we found an effect of 0.14 measured by related posttest items.

The detected comparatively high effect size might follow from what constitutes a related posttest item in the reviewed studies. Some reviewed studies used paraphrases similar to this experiment, yet others used items targeting the same category of information, higher-order items or thematical-related items. In other words, Hamaker (1986) defines relatedness not only *via* paraphrases but also through different item types. However, different effect sizes may be associated with different item types, and the mean effect size in the review aggregates these differences. As the paraphrasing item type is relatively close to repeated posttest items, the effect size may be larger than, e.g., the effect size measured by higher-order items, explaining the comparatively high effect size in our study.

### 6.2. On the Negative Effect of the Adjunct Questions

The second hypothesis, (H2), assuming no difference in the unrelated posttest items for both groups, is not supported by the data. The statistical test for equivalency of the group scores was not significant. Yet, we also did not find the control group to perform significantly superior. Thus, the data neither yields enough evidence to claim inequality or equality of both experimental groups. However, the means and medians of both groups are close, and although the calculated 95% t-confidence intervals are only a rough approximation as the data deviate from the t-distribution, they overlap widely. As a result, although we cannot accept H2 based on the data, we still believe it is a valid hypothesis and assume that the power of the experiment was insufficient to detect an effect. In other words, although H2 is not accepted based on the data, it also should not be rejected based on the data. Accordingly, it is quite possible that the generated questions had no negative impact on the unrelated learning outcome. If there is a negative effect, we assume it will not be huge. This is an important observation because one could theorize about a strong negative effect of the intervention on the unrelated learning outcome. For instance, the intervention may affect learners by increasing their cognitive load with ambiguous, ill-defined questions and may misleads their attention. To summarize the results of the hypothesis tests, the effect of fostering the learning outcome with the AQG is positive, but we cannot completely rule out negative side effects.

### 6.3. Evidence for Why the Adjunct Questions Worked

The explorative data analysis sheds light on why the generated questions increased the related learning outcome. In approximately 50% of the cases, whether a human or a computer asked the question was not apparent. The questions must express certain qualities to fool the learners so frequently. First, they must not contain too many syntactical or grammatical errors. Second, they must have had a minimum of text connection and semantic meaning. Otherwise, we expect learners to conclude that a computer-generated the questions. This argumentation is appealing but has its limits. While the questions' perception as human-written may serve as a proxy for the quality characteristics, it is also confounded by other factors such as the participants' language skills. For example, it has to be noticed that question three (Table 2) was sometimes rated as human, even though it read unnaturally. Furthermore, many generated questions read plausible and contained no obvious mistakes. Consequently, participants may rate them as human-written when asked for a binary decision if the question was computer-generated or human-written. However, this not necessarily indicates that the questions were phrased perfectly. More likely, it only shows that the questions have reached a minimum level of quality to be perceived as human. More nuanced linguistic quality aspects are not measurable using this single evaluation dimension. In summary, we, therefore, take the perceived author ratings as initial evidence that the questions are actively engaging learners. However, on their own, they paint an incomplete picture.

A more complete picture emerges if we combine perceived author ratings, the answers given, and the posttest scores received. Even when learners thought of a question as computer-generated, they usually worked with it. For example, the vast majority of all questions were reviewed and answered by the learners. They derived a suitable response in their own words to the corresponding question. Hence, learners did not need a perfect question resembling a manually authored one to engage with the learning material actively. In other words, computer-generated questions also sufficed in many cases. The scatterplot in Figure 6 is consistent with this observation. It shows that even questions identified as computer-generated often resulted in learning gains. There seems to be no direct relation between related learning outcome and the perceived author of the question. Moreover, the result is in line with similar findings by Van Campenhout et al. (2021), where learners do not prefer human-written questions over computer-generated ones. We suspect computer-generated questions triggered the same cognitive processes as manually authored questions based on these observations. As a result, even questions identified as computer-generated have educational value. However, further evidence is needed before drawing strong conclusions. There are confounding factors not measured currently, such as the learners' perception of what constitutes learning relevant information.

### 6.4. Implications on Educational AQG Research

In the larger context concerning educational AQG, our study provides further evidence that AQG can indeed have a positive impact on learning outcomes. The evidence is important. It shows that when we aim to improve learners' reading comprehension, automatically-generated questions can already make a difference in their understanding. Hence, it might be valuable to incorporate AQG into intelligent textbooks and online reading materials. In those contexts, the generated questions will not replace manually authored questions. Still, they may increase the overall question coverage of text and help learners reach a basic understanding before working with the manually authored questions. Moreover, it generally allows us to derive a larger question pool for self-studying students. Additionally, the experiment opens up a different perspective on expert evaluation. It shows that the questions generated by AQG may not be perceived as human-written but nevertheless improve learning outcomes. If we now look at the expert studies, this is often not taken into account. For instance, system quality is often evaluated by the extent to which they generate natural questions perceivable as human-written. According to our results, however, having questions that are perceived as manually authored may not be necessary to achieve a positive learning effect. Instead, we assume that learners profit from computer-generated questions as long as they meet a minimum linguistic standard, address a concept relevant to learning, and are presented in the appropriate context. What constitutes an appropriate context needs to be explored in future work. For now, we assume that the context anchors the question thematically and helps to disambiguate it. That is, the text context turns an ambiguous question into an unambiguous learning-relevant question. Given these considerations, it may make sense to ask generated questions after reading short text spans. Even if the question is imperfect, learners can anchor the question in what they have read, allowing them to reflect upon the subject matter.

### 6.5. Threats to Validity

We maximized the study's validity by repeatedly testing and revising the reading material, instruction and tests in pilot studies before the actual experiment. We also tried to ensure the comprehensibility and suitability of the texts for the intended target group keeping in mind the usage scenario. Nevertheless, some limitations of the experiment have to be discussed.

First, the sample contains only 48 participants, and although studies exist with similar group sizes per condition (Gustafson and Toole, 1970; Walker, 1974; Rickards, 1976; Callender and McDaniel, 2007), the sample should be considered small. Interestingly, we nevertheless find a significant effect in our statistical analysis despite the limited statistical power given the sample size. Hence, we contribute evidence that AQG systems can foster learning. Yet, due to the small sample, future work should validate our results with larger populations.

Second, due to pandemic conditions, the study was conducted entirely online. Hence, the experimental control of external factors was limited. We addressed this problem explicitly in the instruction to the participants, telling them that they should participate in a quiet, uninterrupted session. Moreover, we added control questions to detect cheating attempts. However, some confounding external factors were likely present during the participation of some subjects in the experiment.

Third, the generalizability might be affected by the reading materials selected. One reason for choosing the respective reading materials was because they were suitable for the generation approach. The generation approach will not work if the materials do not incorporate enough definitions or discuss a few concepts in great detail. Such less definition-heavy textbooks, which focus, e.g., more on the interrelationship of established concepts, are also found in most curricula. While the concrete technical approach of this work will probably not be directly transferable to these texts, we nevertheless assume that some findings generalize. For example, if one has a question generator that works on these texts, it should also be true that not all generated questions must be perceived as human-written to foster learning.

Fourth, according to our operationalization, the related posttest items are inherently connected to the adjunct questions. Therefore, we measure learning progress on those concepts deemed relevant by the question generator. However, these concepts do not necessarily have to be perceived as equally relevant by learners. So in our design, we can't tell if the content learned also fits a typical learning objective a student holds with the reading material. To mitigate this limitation, we deliberately chose a generator basing the question-worthy content selection on didactic premises. In our opinion, it is always relevant for learners to understand the core concepts and their definitions. Yet, this is only an assumption and should be validated in future work. Moreover, when transferring the results to other approaches that, for example, learn the question topics from data, the assumption does not hold, and the limitation should be kept in mind.

Fifth, the participants were mainly not native English speakers. Hence, it is not excluded that native speakers would perceive the questions differently, leading to different conclusions. The direction in which their judgement changes is unclear. Their missing linguistic intuition could lead them to accept more unnatural questions or to complain faster about unknown linguistic constructs. Hence, we cannot exclude possible bias. However, students usually learn how to pose questions in the early stages of language learning. And, as a large majority of involved participants had multiple years of experience in English language learning, their language proficiency was likely sufficient. Moreover, the actual questions were mainly straightforward and did not utilize particularly complex language. Consequently, we believe that advanced non-native speakers can judge their quality. Nevertheless, future studies should validate this assumption with native speakers.

Finally, we would like to mention that asking the learners for the perceived author of the questions may tempt participants to mark at least one question as human-written. Although we explicitly instructed the participants that all questions may be generated, the participants tended not to answer the six binary ratings (computer-generated/human-written) equally. We, therefore, expect some subjects to naively rate a question as human, shifting the results slightly toward human questions.

### 6.6. Conclusions

We have seen that AQG can increase the learning outcome in reading comprehension. Thus, we provide further evidence for the benefits of educational AQG. In contrast to other studies, we focus on learner-centric evaluation. It does not seem necessary for all generated questions to be perceived as human-written to foster learning. Often, participants correctly identified computer-generated questions as such but still had a better learning outcome.

This points to a potential weakness of expert-based evaluations because they usually assume the perception of questions as human-written to be an essential aspect. It remains an open question whether there are adverse effects on the learning outcome of contents not targeted by the AQG. Our data neither confirms adverse effects nor promotes the exclusion of adverse effects. Based on these findings, educational AQG is a promising research direction for scaffolding learners during reading comprehension. Hence, in future work, we plan to look further into the content selection parameters and what constitutes a question-worthy text passage to develop more generalizable AQG approaches.

## Data Availability Statement

The raw data supporting the conclusions of this article will be made available by the authors, without undue reservation.

## Ethics Statement

Ethical review and approval was not required for the study on human participants in accordance with the local legislation and institutional requirements. Written informed consent for participation was not required for this study in accordance with the national legislation and the institutional requirements.

## Author Contributions

TS, AF, and TT collaborated on the study's design and the data analysis. TS assembled the study's initial reading materials and test items and wrote the first draft of the manuscript. AM, AF, and TT analyzed and revised the initial selection of the study's material. TS and AM orchestrated the study's data collection. All authors were involved in writing and revising the manuscript and approved the submitted version.

## Funding

This research was funded by the Bundesministerium für Bildung und Forschung in the project: Software Campus 2.0 (01IS17045), Microproject: Textbook-AQG.

## Conflict of Interest

The authors declare that the research was conducted in the absence of any commercial or financial relationships that could be construed as a potential conflict of interest.

## Publisher's Note

All claims expressed in this article are solely those of the authors and do not necessarily represent those of their affiliated organizations, or those of the publisher, the editors and the reviewers. Any product that may be evaluated in this article, or claim that may be made by its manufacturer, is not guaranteed or endorsed by the publisher.

## Footnotes

^1^OpenStax: Biology2e (4.2); Anatomy and Physiology (5.1).
